# Sprouting of Sorghum (*Sorghum bicolor* [L.] Moench): Effect of Drying Treatment on Protein and Starch Features

**DOI:** 10.3390/foods10020407

**Published:** 2021-02-12

**Authors:** Mia Marchini, Alessandra Marti, Claudia Folli, Barbara Prandi, Tommaso Ganino, Paola Conte, Costantino Fadda, Monica Mattarozzi, Eleonora Carini

**Affiliations:** 1 Department of Food and Drug, University of Parma, Parco Area delle Scienze, 47/A, 43124 Parma, Italy; mia.marchini@unipr.it (M.M.); claudia.folli@unipr.it (C.F.); barbara.prandi@unipr.it (B.P.); tommaso.ganino@unipr.it (T.G.); 2 Department of Food, Environmental, and Nutritional Sciences (DeFENS), University of Milan, Via G. Celoria 2, 20133 Milan, Italy; alessandra.marti@unimi.it; 3 Consiglio Nazionale delle Ricerche, Institute of BioEconomy (IBE), via Madonna del Piano 10, 50019 Sesto Fiorentino, Italy; 4 Dipartimento di Agraria, Sezione Scienze e Tecnologie Ambientali e Alimentari, Università degli Studi di Sassari, Viale Italia 39/A, 07100 Sassari, Italy; pconte@uniss.it (P.C.); cfadda@uniss.it (C.F.); 5 Department of Chemistry, Life Sciences, and Environmental Sustainability, University of Parma, Parma, Italy, Parco Area delle Scienze 17/A, 43124 Parma, Italy; monica.mattarozzi@unipr.it; 6 Centro Interdipartimentale Sulla Sicurezza, Tecnologie e Innovazione Agroalimentare—SITEIA.PARMA, University of Parma, Via Università 12, 43121 Parma, Italy

**Keywords:** sprouting, drying, sorghum, kafirins, starch, physicochemical properties, functionality, nutritional profile

## Abstract

The nutritional and physicochemical properties of sorghum proteins and starch make the use of this cereal for food production challenging. Sprouting is a cost-effective technology to improve the nutritional and functional profile of grains. Two drying treatments were used after sorghum sprouting to investigate whether the drying phase could improve the protein and starch functionalities. Results showed that the drying treatment at lower temperature/longer time (40 °C for 12 h) extended the enzymatic activity that started during sprouting compared to the one performed at higher temperature/shorter time (50 °C for 6 h). An increased protein hydrolysis and water- and oil-holding capacity were found in the flour obtained by the former treatment. Higher protein matrix hydrolysis caused high exposure of starch to enzymes, thus increasing its digestibility, while worsening the technological functionality. Overall, modulating drying conditions could represent a further way, in addition to sprouting, to improve sorghum flour’s nutritional profile.

## 1. Introduction

Sorghum (*Sorghum bicolor* [L.] Moench) is the fifth most important cereal worldwide in terms of production and overall growing area [1]. It is a key dryland food crop cultivated in marginal lands in more than 100 countries. Over 60% of global sorghum production (59.34 million metric tonnes, FAO 2018) comes from developing countries in Africa and Asia. Grown primarily for food by low-income farmers, it offers a staple for over 500 million poor and food-insecure people in around 30 countries in subtropical and semi-arid regions [1]. Elsewhere, sorghum is mostly found in commercial farming for fodder and biofuel [1]. However, its food use in developed countries is increasing, both as a gluten-free cereal for people affected by celiac disease, and for its nutritional potential which encourages industrial development of healthy alternative foods [2,3,4]. Sorghum has huge potential for weight and obesity management due to the relatively low digestibility of its starch. Moreover, its other important nutrients include dietary fiber, fat-soluble and B-vitamins, minerals, and polyphenols [4]. In contrast, well-documented limitations on the food use of sorghum for food production are the poor nutritional and physicochemical properties of its proteins on cooking, which affects starch gelatinization and digestion rates [2,4,5]. These limitations are due to sorghum’s protein organization. Specifically, a high degree of polymerization, extensive disulfide bridges, and the high hydrophobicity of kafirins (representing 77–82% of the proteins in the endosperm [2])—as well as their encapsulation in protein bodies and their strong interaction with tannins and starch—make the use of this cereal for food production a challenge [6].

Biotechnological processing (e.g., fermentation, germination) are used in sorghum-producing countries to overcome these issues [7,8]. Moreover, these processes are widely used at the household level in low-income countries. With regard to germination, it is a proven sustainable approach which triggers synthesis and activation of intrinsic amylases and proteases, which in turn hydrolyze starch granules and proteins into simpler forms, increasing their in vitro digestibility and releasing free sugars and amino acids [9,10]. An impact on the flour’s functional properties has also been documented: protein hydrolysis results in higher solubility, water-holding capacity (WHC) and oil-holding capacity (OHC), foam and emulsion capacity and stability than in native sorghum flour [11,12]. Conversely, worse pasting behavior and changes in textural properties of hydrolyzed starch have also been reported [13].

Although sorghum sprouting has long been investigated, the effect of subsequent drying on the technological and nutritional functionalities of sorghum flour has been poorly explored so far. Since the temperature and time combination is a key parameter in enzymatic activity control [14,15], drying could differently affect and prolong the enzymatic activities during sprouting, conceivably affecting the flour’s nutritional and technological properties.

In the literature, many of the scientific works investigating the issue of germination applied to cereals have carried out a post-germination drying treatment at a temperature between 40 and 50 °C [7,11,13,16,17,18,19]. While on the one hand a drying temperature of 50 °C may be more representative of the conditions adopted in industrial processes performed under controlled conditions of time, temperature and humidity [16,19], on the other, a drying temperature of 40 °C is more representative of the conditions adopted in those contexts where germination is carried out at home and the sprouts are dried in the sun [11,17]. To assess the properties of sorghum flour obtained by sustainable but empirical processes could be of great relevance for the development of sorghum-based food products. Indeed, the outcomes of this research could contribute to market finished products with a tailored and potentially improved nutritional profile, especially for low-income countries where sorghum represents a staple food.

Therefore, this work investigated the effect of drying treatment conducted at 40 °C on sprouted sorghum, in comparison with a drying treatment performed at the higher temperature (50 °C). Specifically, the functionality of starch and proteins were considered to determine whether different drying treatments of the grain might modulate the product functionality.

## 2. Materials and Methods

### 2.1. Sample Preparation

Commercial white sorghum (*Sorghum bicolor* [L.] Moench) kernels were sprouted at an industrial sprouting plant (Bühler AG, Uzwil, Switzerland) under controlled temperature and humidity. The schematic representation of sample preparation is shown in Figure S1.

Sorghum was soaked in water (kernel:water ratio of 1:2) for 16 h at 25 °C and 90% relative humidity (RH) and sprouted for 72 h. Kernels were afterward divided into two aliquots and underwent two different drying treatments. The temperatures chosen for drying were 50 °C, commonly used in industrial sprouting, and 40 °C, to simulate sun-drying in Africa, where sprouting is traditionally performed at home. Therefore, one aliquot was dried for 6 h at 50 °C (SSD50), another for 12 h at 40 °C (SSD40) to obtain the same final moisture content for both processes. Unsprouted (US) and sprouted sorghum were milled using a lab-scale mill (Labormill, BONA, Italy) to produce refined flour, middlings, and bran. Sprouted seed rootlets were recovered before milling and added to the bran. After bran micronization (500 µm), the fractions obtained were reconstituted to wholegrain flour, with the following particle mass distribution: ≈23% particle size >300 µm; ≈30% particle size between 300 and 200 µm, ≈27% between 200 and 100 µm and ≈20% particle size <100 µm. The flour samples were stored at 4 °C until analysis.

### 2.2. Proximate Composition

Protein, lipid, ash, and moisture content were assessed in triplicate by AACC standard methods (46–12.01, 30–25.01, 08–01.01, 44–15.02, respectively [20]), while carbohydrates were determined by difference and the results were expressed as % (g per 100 g) on dry basis (d.b.).

The fiber content (total (TDF), insoluble (IDF) and soluble (SDF)) was determined in duplicate using an enzymatic-gravimetric method according to AOAC Official Methods of Analysis 985.29, 991.42, 993.19, respectively [21].

### 2.3. Protein Characterization and Functionality

#### 2.3.1. Protein Extraction and Fractionation

Sorghum flours were defatted by stirring in 5 vol (*w*/*v*) of petroleum ether for 1 h at room temperature (R/T). After drying, the flour was reground, and petroleum ether extraction was repeated for 30 min. Defatted flour (200× *g*) was extracted following the procedure described by Hamaker et al. [22] and modified by Park and Bean [23]. Briefly, the flour was extracted for 1 h on a shaker at R/T with 0.0125 M sodium borate buffer, containing 2% sodium dodecyl sulfate (SDS) and 2% β-mercaptoethanol (β-ME) (pH 10.0) at a solvent-to-sample ratio of 20:1. The suspension was centrifuged twice at 4637× *g* (6000 rpm) for 25 min (Eppendorf 5810 R, Hamburg, Germany) and the supernatants were pooled. Non-kafirin proteins were precipitated from the total protein extract with 70% ethanol; the mixture was allowed to stand for 2 h with occasional stirring, then centrifuged at R/T for 40 min at 15,585× *g* (11,000 rpm). The supernatant contained the prolamin (kafirin) and non-protein nitrogen fractions was collected; the pellet containing non-kafirin proteins was then solubilized in 2 mL Milli-Q water.

Protein extracts were quantified using the Bradford method [24] and bovine serum albumin (BSA, Sigma-Aldrich, St. Louis, MO, USA) as standard protein. Absorbance values from the samples analyzed were interpolated in a standard curve equation to obtain protein concentration. Twelve determinations were acquired for both kafirin and non-kafirin extracts.

#### 2.3.2. Sodium Dodecyl Sulphate-Polyacrylamide Gel Electrophoresis (SDS-PAGE)

Aliquots of the protein extracts were concentrated using VIVASPIN 500 (Sartorius Biotech, Goettingen, Germany). Amounts of 6 µg of kafirins and 20 µg of non-kafirins were analyzed by SDS-PAGE on 13% polyacrylamide gel in the presence of 0.13% 2-ME using a vertical electrophoresis system (Mini-PROTEAN Tetra System, Bio-Rad, Hercules, CA, USA). A broad range protein molecular weight standard (10–250 kDa) was used (BioRad, Richmond, CA, USA). Gels were stained with 0.25% Coomassie Brilliant Blue R and the images digitized with a ChemiDoc MP Gel Imaging System scanner (Bio-Rad, Hercules, CA, USA).

### 2.4. Amino Acid Analysis

Sorghum flour (500 mg) underwent acid hydrolysis and derivatization with AccQ Tag (Waters, Milford, MA, USA) as described in Anzani et al. [25]. All samples and standard solutions were analyzed using an ultra-performance liquid chromatography coupled to electrospray ionization mass spectroscopy (UPLC/ESI-MS) system as described by Buhler et al. [26]. The ratio of essential amino acid (EA) to total amino acid (TA) was calculated (EA/TA, %).

### 2.5. Water-Holding Capacity (WHC) and Oil-Holding Capacity (OHC)

WHC and OHC were measured in triplicate according to Marchini et al. [27]. Briefly, 100 mg of flour was mixed with 1 mL of distilled water (WHC) or sunflower oil (OHC), shaken with a vortex for 30 s, and then allowed to rest at R/T for 30 min. Mixtures were centrifuged at 2061× *g* (4000 rpm) for 20 min and the supernatant was then decanted. WHC and OHC were calculated as the ratio between the grams of water or oil per gram of solid.

### 2.6. Starch Characterization and Functionality

#### 2.6.1. Total Starch (TS), Resistant Starch (RS), Digestible Starch (DS), Amylose Content

Total starch (TS), resistant starch (RS) and digestible starch (DS) were determined using a Megazyme Resistant Starch Assay Kit (K-RAPRS, Megazyme International Ireland Ltd., Wicklow, Ireland) following AACC standard method no. 32-40.01.

Amylose content was quantified in triplicate using the Megazyme amylose/amylopectin assay procedure (K-AMYL 06/18 commercial kit, Megazyme International Ireland Ltd., Wicklow, Ireland) following the manufacturer’s protocol. The amylose/amylopectin ratio was then calculated for each sample.

#### 2.6.2. α-Amylase Activity, Pasting and Thermal Properties and Swelling Power (S_P_)

α-amylase activity was determined according to AACC standard method no. 22-02.01, using the Megazyme Amylase Assay Procedure (K-CERA, Megazyme International Ireland Ltd., Wicklow, Ireland).

Pasting properties of flours were measured in triplicate using a Micro-Visco-Amylograph device (MVAG, Brabender GmbH & Co. KG, Duisburg, Germany) as described by Marti et al. [19], using a 1 mM aqueous AgNO_3_ solution instead of distilled water to inhibit α-amylase activation during analysis. Briefly, flour (12 g) was dispersed in 100 mL of a 1 mM aqueous AgNO3 solution and stirred at 250 rpm. Pasting properties were determined applying the following temperature profile: heating from 30 to 95 °C at a rate of 3 °C/min, holding at 95 °C for 20 min, cooling from 95 to 30 °C at a cooling rate of 3 °C/min, and holding at 30 °C for 1 min.

The pasting temperature (temperature at which gelatinization begins, °C), peak viscosity (maximum viscosity value of the slurry during gelatinization, Brabender Units, BU), peak temperature (temperature at which peak viscosity occurs, °C), final viscosity (viscosity of the slurry at the end of the test, BU), breakdown (difference between peak viscosity and minimum viscosity during the holding period, BU) and setback (difference between peak and final viscosities, BU) were calculated from the pasting curve.

Thermal properties of flours were measured in triplicate using a differential scanning calorimeter (DSC Q100 TA Instruments, New Castle, DE, USA, USA), calibrated with indium (melting point: 156.6 °C, melting enthalpy: 28.71 J/g) and mercury (melting point: −38.83 °C, melting enthalpy: 11.44 J/g), as described by Marchini et al. [27]. Briefly, flour and distilled water were mixed in a ratio of 1:3 *w*/*v* and left to equilibrate overnight at R/T. An aliquot of the water–flour suspension (5–10 mg) was placed in stainless steel pans (Perkin Elmer, USA) hermetically sealed, quench cooled to 30 °C and then heated to 120 °C at 5 °C/min, using an empty pan as reference. The enthalpy (ΔH, J g^−1^), onset (T_on_, °C), peak (T_p_), and offset (T_off_, °C) temperatures of the observed transitions were obtained from heat flow curves using Universal Analysis Software, Version 4.5A (TA Instruments, New Castle, DE, USA).

S_P_ was measured as described previously by Marchini et al. [27]. Briefly, flour suspensions (2% *w*/*v*) were heated in a water bath at 60, 70, 80 or 90 °C for 1 h and cooled at 30 °C for 30 min. The samples were centrifuged at 8243× *g* (8000 rpm) for 20 min and the precipitates were weighed. S_P_ was calculated as the ratio between sediment and dry sample weights.

Determinations were in triplicate.

### 2.7. Microstructure

The size and distribution of sorghum flour sample cells were examined using optical microscopy (DM 4000B, LEICA, Wetzlar, Germany). Flour particles were stained using toluidine blue (0.1%); 6 slides per flour were analyzed. Images of cells (6) and cell agglomerates (18) were observed at 20× and 1.25×, respectively, and acquired by a camera (Leica DMC2900, Wetzlar, Germany). Cell aggregate areas were obtained using imaging analysis software (Leica, IM50 Version 4.1, Germany). Agglomerates were arbitrarily divided into four dimensional classes, as follows: Class I: 1–50,000 μm^2^; Class II: 50,001–100,000 μm^2^; Class III: 100,001–150,000 μm^2^; Class IV: >150,000 μm^2^, and the average number of representative agglomerates for each class calculated. The number of cells per agglomerate was calculated by dividing the average aggregate area by the mean cell area.

Ultrastructural analysis of flours was performed with an Environmental Scanning Electron Microscope Quanta™ 250FEG ESEM (FEI, Hillsboro, OR, USA). The samples, after fixing to a stub with carbon double-sided tape, were directly analyzed in low vacuum mode (pressure chamber at 70 Pa) with a beam accelerating voltage of 5 kV. Magnification ranges of ESEM micrographs (≥10 micrographs acquired) were 3000–6000×.

### 2.8. Statistical Analysis

To determine significant differences between samples, data were analyzed by one-way analysis of variance (ANOVA) followed by Duncan’s post-hoc test at a 0.05 significance level. Analyses used SPSS Statistical Software (Version 25.0, IBM SPSS Inc., Chicago, IL, USA).

## 3. Results and Discussions

### 3.1. Proximate Composition

Proximate composition for unsprouted sorghum flour (US) agreed with the literature [6], differing significantly (*p* ≤ 0.05) from that of the sprouted samples (Table 1). Moisture content was comparable in sprouted samples, and higher than US. Additionally, the data revealed a slight increase in protein content for SSD40 only (+6.2%), while SSD50 did not differ significantly from US. Moreover, the sprouting significantly (*p* ≤ 0.05) decreased the fat (−12.4% and −17.3% for SSD50 and SSD40, respectively), ash (−3.7% and −1.5% for SSD50 and SSD40, respectively), and carbohydrate (−0.9% and −1.9% for SSD50 and SSD40, respectively) contents compared to US, in agreement with Lemmens et al. [8], who reviewed the effect of sprouting on cereals. Changes in proximate composition were always greater in SSD40 than in SSD50. The ~6% increase in protein showed by SSD40 compared to US may be due to the release of a much greater amount of free amino acids through protein synthesis in the embryo [10]. A relative difference in protein content between sprouted and unsprouted cereals of less than 10% can be considered negligible [8]. The significant decrease (*p* ≤ 0.05) in lipid content has to be related to hydrolysis of fat components into fatty acids and glycerol caused by the lipase activity activated during sprouting [12], which leads them into the metabolic gluconeogenesis pathway.

Conceivably, the higher fat content found in SSD50 flour may be caused by a higher lipase activity inactivation consequent to the higher drying temperature compared to SSD40, therefore closer to the lipase inactivation range (60–80 °C) [28].

A decrease in macronutrients on sprouting has also been reported by Afify et al. [10]. However, the different time/temperature combination of the drying treatment which the sprouted grain underwent, differently affected the enzymatic and metabolic processes activated during sprouting, causing the observed slight but significant differences in the proximate composition of the flours.

Sprouting processes did not affect either TDF or IDF (which comprises more than 95% of TDF) content of sorghum to any great extent (Table 1). The results suggest that the processes used did not significantly modify the cell walls, as verified by optical microscopy images (see Section 3.4); the IDF found in the outer layer of the grain is conceivably not prone to much degradation during sprouting and heating. Similar dietary fiber composition as a function of sorghum sprouting can be found in the literature [6].

### 3.2. Protein Characterization and Functionality

#### 3.2.1. Protein Extraction and Fractionation

The protein extraction procedure used in this study was chosen on the basis of the authors’ suggestion that a reasonable—and simpler—classification of sorghum proteins would be to divide them into kafirin and non-kafirin groups. This classification reflects the homogeneous nature of the kafirin storage prolamins, as opposed to the heterogeneous group of non-kafirins (namely, albumins, globulins and glutelins), involved in cellular functions [2]. This procedure was effective for the extraction and fractionation of both kafirins and non-kafirins, as confirmed by colorimetric determinations performed on protein extracts. Specifically, protein concentrations (given by the Bradford method) in the kafirin extracts were 0.29 ± 0.04 µg/µL, 0.33 ± 0.02 µg/µL and 0.48 ± 0.06 µg/µL for US, SSD50 and SSD40, respectively, while protein concentration in non-kafirin extracts was 4.66 ± 0.62 µg/µL, 7.99 ± 0.75 µg/µL and 6.67 ± 0.38 µg/µL for US, SSD50 and SSD40, respectively.

#### 3.2.2. SDS-PAGE

**Figure 1:**
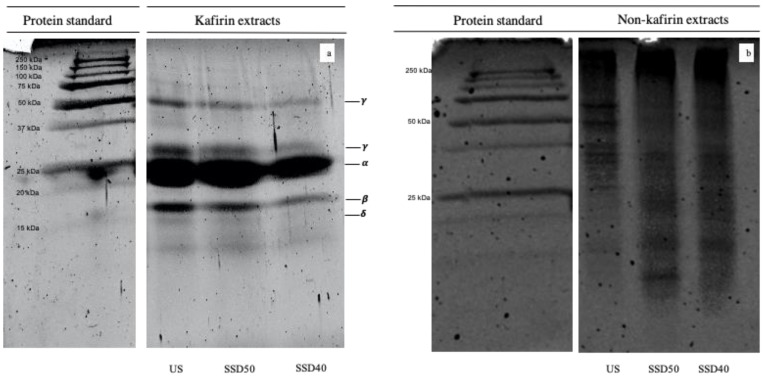
Sodium dodecyl sulfate polyacrylamide gel electrophoresis (SDS-PAGE) of kafirin (**a**) and non-kafirin (**b**) fractions of sorghum flours. US, unsprouted sorghum flour; SSD50, flour from sprouted sorghum dried at 50 °C for 6 h; SSD40, flour from sprouted sorghum dried at 40 °C for 12 h.

The kafirins and non-kafirins extracted from sorghum flours were evaluated using SDS-PAGE, as shown in Figure 1.

The electrophoretic system showed a typical reduced kafirin electrophoretic profile (Figure 1a), characterized by the absence of any lanes generated by high molecular weight (HMW) aggregates and >50 kDa oligomers. Indeed, reducing conditions caused an S-S bond cleavage which disrupts the oligomers, leading to the appearance of low molecular weight constituents [29]. Accordingly, Figure 1a illustrates three main fractions with a molecular weight (Mr) of 16–18 kDa, 23–25 kDa and 27–28 kDa, corresponding to β-, α-, and γ-kafirin, respectively [2,29]. The lane generated by α-kafirin predominated and was created by the overlapping of two bands generated by two subunits (α_1_- and α_2_-kafirin) with slightly different mobility [29]. Furthermore, the visible minor band of Mr at about 49 kDa (Figure 1a) had previously been attributed to an unreduced oligomer of γ-kafirin [30], while from among the fainter low molecular weight lanes identified at Mr ≤ 15 kDa, Belton et al. [30] detected the 14 kDa δ-kafirin component.

As expected, a decrease in prolamin electrophoretic lane intensities was observed upon sprouting (Figure 1a), especially in those generated by β-, and γ-kafirin. This evidence is consistent with the literature and has been related to prolamin hydrolysis [9,31]. Given that kafirin protein bodies consist in an outer “shell” composed mainly of crosslinked β- and γ-kafirins and a core mainly containing α-kafirin [2], β- and γ-kafirins are the first proteins to degrade during sorghum sprouting. Since this degradation begins from the surface, the β- and γ-kafirins breakdown may be explained by their peripheral location [31]. The observed decrease in prolamin electrophoretic lane intensities was more pronounced in the SSD40 kafirin extract (Figure 1a), which may be due to a higher prolamin hydrolysis than that in SSD50 during sprouting.

The greater differences in SSD40 may have been caused by the drying conditions used: 40 °C and 50 °C proved to be close to the optimum temperature range for cereal protease activity [15], while the SSD40 prolonged drying time may have caused not only a failing enzyme inactivation effect, but also prolonged enzymatic activity over time.

The electrophoretic analysis of non-kafirin protein fraction (Figure 1b) showed a profile characterized by a broader number of bands distributed throughout the electrophoretic gel, attributable to HMW aggregates, oligomers and monomers of albumins, globulins and glutelins, which constitute around 10% of wholegrain sorghum flour proteins [22] in agreement with data in the literature [22,32].

Compared to US, small changes were observed in the SDS-PAGE analysis of non-kafirin extracts on sprouting (Figure 1b). SSD50 and SSD40 non-kafirin extracts exhibited a slight decrease in the intensity of lanes characteristic of HMW aggregates in favor of an increase in the intensity of oligomer and monomer bands (≤50 kDa), confirming the effect of proteolytic activity [32].

### 3.3. Amino Acid Analysis

The amino acid profile of unsprouted and sprouted sorghum (Table 2) was found to be comparable overall with those reported in the literature [33].

With a value higher than 0.5 g/100 g flour, the major amino acids are leucine, phenylalanine, valine (essential amino acids, EA), alanine, proline and aspartic and glutamic acids (non-essential amino acids, NEA). Moreover, a favorable amino acid balance determined by high levels of EA (EA/TA % ~43%) was found, confirming sorghum as a high-value product from a nutritional point of view [33].

As expected, the percentage of hydrophobic amino acids (AA) (isoleucine, leucine, methionine, phenylalanine, valine, alanine, hydroxyproline and proline) accounted for ~49% of the total, thus confirming the hydrophobic characteristics of sorghum proteins, especially kafirins [2]. The second most abundant AA were the acidic ones (aspartic and glutamic acid) which represented ~28% of the total, followed by the uncharged polar AA (glycine, serine, threonine, tyrosine, and cysteine) accounting for ~16.5% and the basic amino acids (lysine, arginine, histidine) representing 7%. An increase in several AA was observed after sprouting (Table 2) with values in agreement with the literature [10], which has also reported an improved amino acid profile on sprouting. During the metabolic processes activated during sprouting for the developing embryo, AA may be produced in excess of requirements and tend to accumulate in the free amino acid pool [10]. Amino acids, especially those which increase during sprouting, are involved in such fundamental physiological plant activities and metabolic pathways, as protein and secondary metabolites synthesis, which in turn play a vital role in plant growth, regulation of plant metabolism, resistance to abiotic, water and osmotic stresses, increases in germinability as precursors of growth factors, development of new tissues, and hormonal-like activities [34].

In any case, it was interesting to observe that changes to AA profiles after sprouting were drying-treatment-dependent. The total AA content significantly increased only in SSD40 (+5.8%) compared to US, while SSD50 showed no significant differences compared to US. Among the EA, the isoleucine content of SSD40 showed a greater increase (5.1%) than SSD50 (3.8%), while the change in lysine was similar to that of isoleucine except that the amplitude was larger (8.8% and 4.1% for SSD40 and SSD50, respectively). The increase in methionine and cysteine content measured in SSD40 was around six times higher than in SSD50 (11.8% vs. 3% and 5.3% vs. 1.1%, respectively). As for the EA, SSD40 showed a twofold increase in aspartic acid compared to SSD50 (20.7% vs. 12%), proline (16.1% vs. 9%) and serine (6.2% vs. 2.3%). In SSD40, also the alanine content increased (3.6%) compared to US, while SSD50 showed a decrease (2%). Therefore, the drying conditions may also have influenced the amino acid composition of the flour. It has been hypothesized that the treatment carried out at 50 °C for 6 h (SSD50) may also have caused higher AA degradation than that carried out at a lower temperature (40 °C for 12 h, SSD40).

### 3.4. Water-Holding Capacity (WHC) and Oil-Holding Capacity (OHC)

The US sample showed a WHC (Table 1) within the range previously identified in the literature [35]. Sprouting significantly increased WHC (*p* ≤ 0.05) only in the case of SSD40.

Likewise, sprouting processes significantly increased the OHC of sorghum flours (*p* ≤ 0.05), with SSD40 showing the higher values. Data were within the range identified by Elkhalifa and Bernhardt [11] for sprouted sorghum.

The impacts of sprouting on WHC and OHC have already been well documented [11,12,35] and also related to changes in protein functionality due to the proteolytic enzymes activated during sprouting, which give rise to an improved capacity to retain water and fat globules [8,11,12]. Specifically, changes in hydration properties may be due to increased polar groups in proteins and polysaccharides upon sprouting. The exposure of part of the water binding site on the side chain groups of proteins seemingly leads to an increase in sites which interact with water and a corresponding increase in WHC in sprouted samples [12].

Similarly, a higher OHC appears to be due to the dissociation and partial unfolding of polypeptides which exposes the hydrophobic amino acids sites while favouring hydrophobic association of peptide chains with lipid droplets [12]. Therefore, the decreased fat content in the sprouted samples may have been due to the ability of proteins to absorb more oil. Consequently, the higher WHC and OHC recorded for SSD40 may be related to a more intensive protein hydrolysis in this sample, as discussed above (Section 3.2).

### 3.5. Starch Characterization and Functionality

#### 3.5.1. Total Starch (TS), Resistant Starch (RS), Digestible Starch (DS), Amylose Content

The effect of sprouting on sorghum starch structure was assessed by RS, DS and amylose content determinations. The starch content of native sorghum flour was 73.7 g/100 g d.b. (Table 1), of which 9.24% was RS, in line with the literature [3,36]. Furthermore, in native sorghum flour, DS was found to be 90.8% of TS. As expected, a decrease in TS (~5%) occurred in both SSD50 and SSD40 (Table 1), due to the starch-degrading enzymes activated and de novo synthesized during sprouting. An increase in DS [8] with a decrease in RS upon sprouting was found previously in cereals [37]. Notably, the percentage of RS out of TS content decreased to 6.6% in SSD50 and 6.3% in SSD40, showing that the latter had slightly higher starch digestibility. Indeed, the percentage of DS with respect to TS increased slightly more in SSD40 than in SSD50 (93.7 and 93.5%, respectively).

The increase in starch digestibility found in the sprouted samples may be due to protein hydrolysis and breakage of the disulfide bond cross-linking involving kafirins in the protein matrix, thereby rendering the starch granules more susceptible to enzymatic action [3]. Furthermore, in accordance with Lemmens et al. [8], starch digestibility may have increased upon sprouting because of the higher content of enzymatically damaged starch granules, thin cell walls, and more readily available sugars. An increase in starch digestibility makes sprouted sorghum flour suitable to produce food for infants, the elderly, or undernourished people who require a readily available source of energy [8], or to fortify staple foods (e.g., flatbread) for developing countries to potentially increase their nutritional value. Overall, as shown by the higher DS found in SSD40 than SSD50, also drying treatment after sorghum sprouting can further improve the starch digestibility of the product.

Amylose content (%) and amylose/amylopectin ratios of the flours are reported in Table 1. Unsprouted sorghum flour data agreed with the literature [4]. Sprouting increased amylose content (therefore, the amylose/amylopectin ratio) especially for SSD40 treatment. Indeed, increased amylose content was observed in both sprouted samples (+17% and +62% for SSD50 and SSD40, respectively), even if US and SSD50 did not differ statistically. The increase in amylose content and amylose/amylopectin ratio may be due to the preferential hydrolysis of amylopectin chains and cleavage of its long chain branches by amylases de novo synthesized and activated during sprouting [14]. The higher amylose/amylopectin ratio found for SSD40 compared to SSD50 documented the higher, more extensive hydrolytic activity which SSD40 underwent, conceivably related to increased starch accessibility thanks to the higher protein matrix degradation observed in this sample, as discussed previously.

#### 3.5.2. α-Amylase Activity, Pasting and Thermal Properties, Swelling Power (S_P_)

As expected, the sprouted samples featured much more enzymatic activity than US, with no significant differences between the two sprouted samples (Table 1). Data confirmed the de novo synthesis and accumulation of α-amylase in scutellum and aleurone cells during sprouting, leading to partial hydrolysis of starch into sugars which are an energy source for the developing embryos [8]. The comparable values between the two samples suggest that drying was able to promote α-amylases activity over the treatment time in the same way, possibly due to the optimal temperature ranges for enzymatic activity [14] in both drying treatments. Despite this, as shown previously, the starch fraction was differently affected by drying as a result of a different protein matrix degradation which conceivably affected starch accessibility.

**Figure 2:**
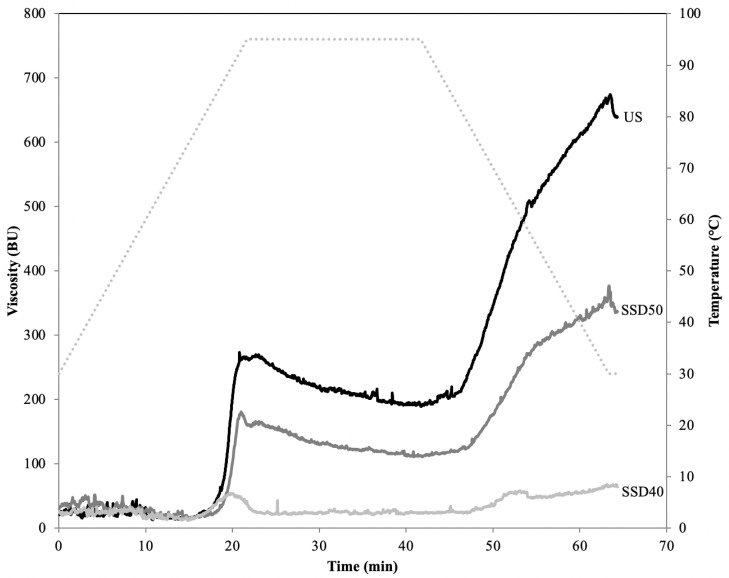
Pasting properties of sorghum flour samples measured by means of a Micro-Visco Amylograph. US, unsprouted sorghum flour; SSD50, flour from sprouted sorghum dried at 50 °C for 6 h; SSD40, flour from sprouted sorghum dried at 40 °C for 12 h.

The presence of endogenous α-amylase strongly impacted the samples’ pasting profile (Figure 2), since the temperature profile of the analysis caused enzyme activation and thus starch hydrolysis [38]. Accordingly, amylases were inhibited with a 1 mM aqueous AgNO_3_ solution to understand any pasting profile changes due to experimental variables [38].

The pasting temperature recorded for the US sample was 78.8 ± 0.3 °C; SSD50 showed a significantly (p ≤ 0.05) higher value, indicating the prerequisite of higher temperatures to reach full granule swelling, while a decrease in SSD40 was found (Table 1). Since it is the starch structure which governs the initial gelatinization point and the range the gelatinization occurs over, pasting temperature shifts were apparently caused by modification of starch granule structure, as reported elsewhere [13].

As expected, a peak viscosity decrease occurred upon sprouting, with SSD40 showing the greater decrease (Table 1 and Figure 2). This is due to several factors, including starch degradation, debranching to simpler units, and changes in proteins and fatty acids [5,39]. The marked viscosity loss in the samples was accompanied by a peak temperature decrease, supporting the assumption that extensive starch breakdown caused by endogenous enzymes had occurred during sprouting, especially during SSD40 treatment. Correspondingly, sprouting also provoked a final viscosity decrease, with SSD40 showing the lowest value. Additionally, both samples showed a lower breakdown value than US, suggesting a starch heat-stability increase [13].

The setback value—reflecting the retrogradation tendency of amylose in starch paste—decreased in both sprouted samples, suggesting a decrease in starch retrogradation ability compared to US. During sprouting, the outermost branches of amylopectin are hydrolyzed by α-amylase and are thus no longer able to form large amylopectin crystals. These small crystallites cannot form a three-dimensional network capable of promoting a major increase in viscosity during cooling [19]. This trend could prove of interest to the food industry and bakery sector given that low setback values indicate a low rate of starch retrogradation and syneresis.

Overall, sprouting resulted in a significant decrease (p ≤ 0.05) in viscosity during the heating and cooling phases as a consequence of degradative processes on starch phase activated by the sprouting. The different pasting behavior between SSD50 and SSD40 may be attributed to the different magnitude of enzymatic activity inhibition that occurred during drying. SSD50 still had the ability to form a gel below 95 °C, which is interesting with a view to formulating products using sprouted sorghum with improved nutritional and technological properties. Indeed, SSD40 did not show a typical pasting profile despite the addition of AgNO_3_ as inhibitor; in particular, there was no real viscosity peak and the curve remained flat throughout the analysis as previously observed on other cereals [13]. The lower drying temperature condition could therefore find application in food formulation where extensive gelatinization of starch is not required (e.g., flatbread) with the advantage of providing improved starch digestibility.

The swelling power (S_P_) of the flours was determined at four different heating temperatures to better reflect the structural changes to starch functionality as a result of the sprouting treatments (Table 1). As expected, S_P_ showed a continuous increase as the temperature rose in all the samples in agreement with previous studies [39]. Moreover, the data revealed that S_P_ significantly decreased as a consequence of the sprouting, as previously reported on other cereals [12]. Among the sprouted samples, SSD40 showed the lowest S_P_ values at all temperatures. At 60 °C, US and SSD50 presented a comparable S_P_ value, while SSD40 had a significantly lower S_P_ (p ≤ 0.05). Nevertheless, at 70 °C, the S_P_ of SSD40 increased to become comparable with that of the other two flours. A more substantial increase in S_P_ in all samples when the temperature began to reach ~80 °C was found, as expected [40], while a further increase in temperature (90 °C) did not significantly modify the flours’ S_P_.

It is widely accepted that amylopectin is the primary component responsible for starch S_P_ and is also the component mainly responsible for the formation of the starch crystalline structure [40]. The reduction in S_P_ may be due to changes in both starch content (Table 1) and structure (i.e., amylose/amylopectin ratio) due to enzymatic activities. In addition, degradation by α-amylase causes accumulation of dextrin, oligosaccharides and fermentable sugars which have no S_P_, thereby interfering with the starch formation of more compact gels [12]. Overall, the difference in S_P_ found between SSD40 and SSD50 may be attributed to the different degree of starch and amylopectin degradation during sprouting and drying.

**Figure 3:**
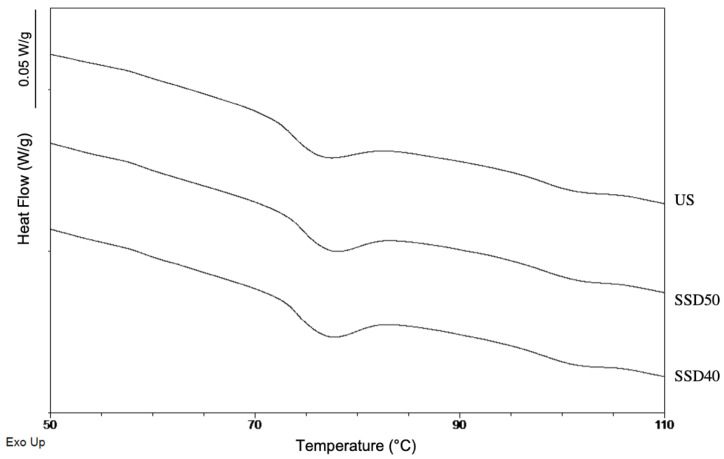
Representative differential scanning calorimeter (DSC) thermograms of US, SSD50 and SSD40 flours in the range 50–110 °C. US, unsprouted sorghum flour; SSD50, flour from sprouted sorghum dried at 50 °C for 6 h; SSD40, flour from sprouted sorghum dried at 40 °C for 12 h.

DSC representative thermograms and thermal properties of the flours are shown in Figure 3 and Table S1, respectively.

Two endothermic events were evident for all flours (Figure 3), with the main thermal transition found between ~58 and ~91 °C, corresponding to starch gelatinization. Thermograms of sprouted samples also showed a minor endothermal event between ~58 and ~65 °C, likely related to a small fraction of starch gelatinizing at a lower temperature.

Average gelatinization temperatures agreed with those in the literature, while the gelatinization enthalpy was lower [4,39] possibly due to genetic and/or environmental factors [4].

As seen in Table S1, a significant (p ≤ 0.05) increase in T_on_, a decrease in T_off_ and a decrease in enthalpy occurred upon sprouting with no significant differences between the two sprouted samples.

The third endothermic event was found in the temperature range ~94–108 °C (Figure 3) similar to that reported in the literature and related to the melting of amylose–lipid complexes [4]. The slightly higher melting enthalpy of amylose–lipid complexes recorded by SSD40 may be related to its higher amylose content (Table 1). Overall, in all the endothermic peaks identified, the thermal parameters showed no significant variations as a result of the drying treatment. These results suggested that the experimental variables did not affect the flours’ thermal properties at a mesoscopic level in conditions of excess water. Conversely, as shown previously, the same experimental variables significantly affected pasting properties at the macroscopic level when measured with an empirical approach.

### 3.6. Microstructure

Optical microscopy was used to investigate the effect of sprouting and drying on cell aggregates and cell wall integrity (Figure S2). Indeed, during cereal sprouting and subsequent drying, an enzymatic and/or thermal degradation of the cell wall’s non-starchy polysaccharide components may occur [41,42].

For all flours, the cells were sorted into different-sized aggregates (Figure S1b,d,f) with a heterogeneous dimension distribution (Figure S1a,c,e). For all flours, the majority of cell aggregates (~60%) were small in size (1–50,000 μm^2^), ~20% of the agglomerates were in the second dimensional class, ~11% were in class III, while only 9% of the agglomerates had an area >150,000 μm^2^. Figure S2 (right) shows the average number of cells per agglomerate, calculated by dividing the area of each aggregate by the calculated mean area of the cells (8598 μm^2^). The number of cells per agglomerate belonging to different classes did not differ among the samples, indicating that neither the sprouting nor the drying treatment had caused degradation of the pectin-rich cell wall fractions in any of the cases. The absence of significant differences in the cell morphology in different samples indicates that the impact of the various treatments on the sorghum was negligible.

**Figure 4:**
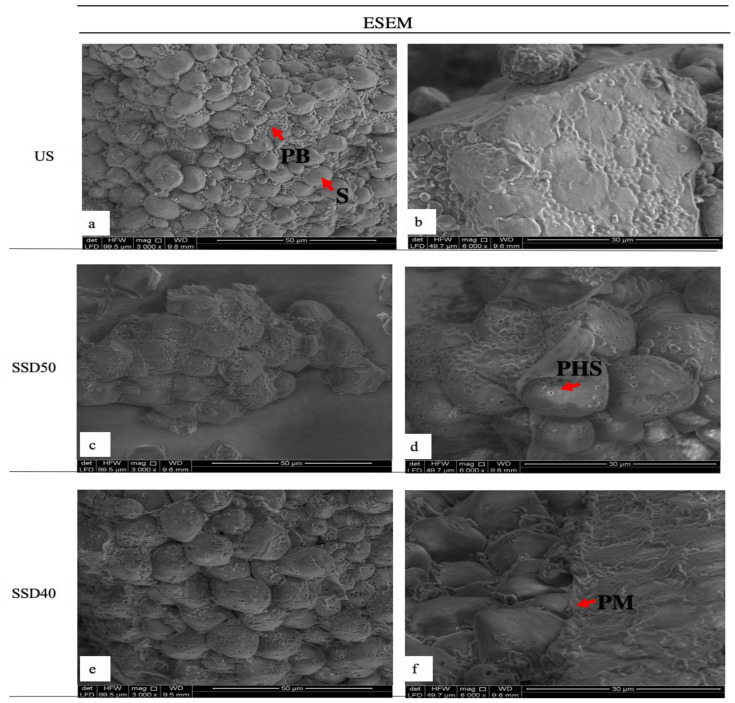
Morphological observations of sorghum flours acquired with environmental scanning electron microscopy (ESEM). Micrographs of US, SSD50 and SSD40 flours were acquired with a magnification of 3000× (**a**,**c**,**e**) and 6000× (**b**,**d**,**f**). US, unsprouted sorghum flour; SSD50, flour from sprouted sorghum dried at 50 °C for 6 h; SSD40, flour from sprouted sorghum dried at 40 °C for 12 h; S, starch granule; PB, protein body; PHS, partially hydrolyzed starch; PM, protein matrix.

Figure 4 shows ESEM images of sorghum flours.

US (Figure 4a,b) was mainly constituted of compacted and intact starch granules ranging from approximately 15 to 25 µm in size, polygonal in shape, surrounded by spherical protein bodies of around 1 µm in diameter, and enclosed in a compact protein matrix to which protein bodies were attached (Figure 4a). The starch and protein dimensions and micrographs were similar to those in previously published works [4,9]. The ESEM images of the sprouted flours (Figure 4c–f) revealed that sprouting affected the sorghum ultrastructure to a different extent depending on the type of drying treatment used. In both SSD50 and SSD40, the proteolytic activity which occurred during sprouting determined a partial degradation of the proteinaceous coating (Figure 4d,f) leading to a release of starch granules and protein bodies. In addition, the protein bodies seemed more detached from the starch granules if compared with US flour. In SSD50, the starch granules appeared eroded in the sites where the protein bodies were located (Figure 4c,d), while in SSD40 the protein bodies seemed no longer visible (Figure 4e). Additionally, partial hydrolysis of the starch granules was visible in both sprouted samples but appeared more substantial in SSD40 (Figure 4d,e), confirming the data on protein and starch enzymatic degradation, and suggesting that this sample was conceivably more affected by amylolytic activity.

## 4. Conclusions

Two drying treatments were selected and used after sorghum sprouting. The effects on protein and starch features were analyzed to determine if the drying treatment could contribute to an improvement in the final product functionality.

Sprouting caused significant changes in the flours’ properties, improving their nutritional profile, confirmed by the increase in total amino acid content. Moreover, increased WHC and OHC and worsened starch gelatinization underscore that sprouting also affected protein and starch flour functionality.

Significant differences in the flours’ functional and nutritional properties were also found as a function of the drying temperature. The treatment performed at a lower temperature (40 °C) and longer time (12 h) seemed to have favored extended enzymatic activity over time. Indeed, a higher protein hydrolysis was found in this sample resulting in increased capacity to hold water and fat globules. Additionally, the higher protein matrix deterioration may have caused greater starch exposure to the enzymes leading to higher hydrolysis and increased digestibility. Nevertheless, this worsened starch functionality.

Overall, the drying treatment performed on the sprouts could represent an effective, sustainable method, in addition to the sprouting phase in sensu stricto, to improve the nutritional profile of sorghum. Further studies are needed to investigate the effect of the two treatments on the content and accessibility of sorghum micronutrients and bioactive compounds. This insight could be useful to provide a broader overview on the effective potential of drying treatment performed at 40 °C to improve a product’s nutritional properties. Moreover, the study of additional post-sprouting drying conditions may be of interest to provide further processing indications useful to modulate nutritional and technological functionalities of final products.

From a technological point of view, these flours from sprouted sorghum could be used where high starch performance is not required, e.g., unleavened bread. Future research is also needed to further investigate the molecular and rheological properties of derived sprouted sorghum flour-based dough, in order to optimize and use sprouting—an inexpensive cost-effective technology—to market finished products with an improved nutritional profile. Overall, the outcomes of the sprouting process performed with a drying treatment at a temperature of 40 °C may be of great relevance for those countries where sorghum is a staple food, sprouting is performed at household level and the sprouts are dried in the sun.

## Figures and Tables

**Table 1:** Proximate composition, physico-chemical and pasting properties of unsprouted and sprouted sorghum flours. Compositional data are expressed as g/100 g dry basis (d.b.). In brackets, soluble dietary fiber (SDF) refers to g/100 g d.b. total dietary fiber (TDF); resistant starch (RS) and digestible starch (DS) refer to g/100 g d.b. total starch (TS).

	US	SSD50	SSD40
Protein (g/100 g d.b.)	11.27 ± 0.31b	10.98 ± 0.48b	11.97 ± 0.00a
Fat (g/100 g d.b.)	3.71 ± 0.03a	3.25 ± 0.00b	3.07 ± 0.02c
Moisture (g/100 g w.b.)	11.31 ± 0.15b	12.69 ± 0.04a	12.67 ± 0.03a
Ash (g/100 g d.b.)	1.35 ± 0.02a	1.30 ± 0.00c	1.33 ± 0.00b
Carbohydrates (g/100 g d.b.)	85.25 ± 0.12a	84.48 ± 0.53b	83.63 ± 0.07c
Total Dietary Fiber (TDF, g/100 g d.b.)	7.4 ± 0.67	7.1 ± 0.5	7.4 ± 0.7
Insoluble (IDF, g/100 g d.b.)	7.3 ± 0.8 (98.65)	7.1 ± 0.5 (98.59)	7.1 ± 0.85 (95.95)
Soluble (SDF, g/100 g d.b.)	<LOQ	<LOQ	<LOQ
WHC (g/g)	1.51 ± 0.05b	1.52 ± 0.08b	1.68 ± 0.05a
OHC (g/g)	0.95 ± 0.01b	1.00 ± 0.07ab	1.05 ± 0.00a
Total Starch (TS, g/100 g d.b.)	73.7 ± 0.0a	69.9 ± 0.1b	69.1 ± 0.2c
Digestible (DS, g/100 g d.b.)	66.9 ± 0.0a (90.8)	65.3 ± 0.0b (93.5)	64.8 ± 0.3c (93.7)
Resistant (RS, g/100 g d.b.)	6.8 ± 0.0a (9.2)	4.6 ± 0.1b (6.6)	4.3 ± 0.2c (6.3)
Amylose (%)	29 ± 3b	34 ± 4b	47 ± 0a
Amylose/Amylopectin ratio	0.41	0.51	0.88
α-amylase activity (CU/g)	0.06 ± 0.01b	22.60 ± 0.85a	20.64 ± 0.88a
Pasting properties (MVAG Test)			
Pasting temperature (°C)	78.7 ± 0.3b	80.9 ± 1.1a	76.5 ± 0.1c
Peak viscosity (BU)	269.5 ± 3.5a	187.0 ± 6.0b	92.5 ± 5.5c
Peak temperature (°C)	93.7 ± 1.3a	92.2 ± 0.8ab	89.9 ± 1.9b
Final viscosity (BU)	643.5 ± 4.5a	335.5 ± 1.5b	105.0 ± 11.0c
Breakdown (BU)	80.0 ± 2.0a	74.5 ± 6.5b	47.5 ± 6.5c
Setback (BU)	482.5 ± 5.5a	247.50± 15.5b	59.0 ± 17.0c
S_P_ (g/g)			
60 °C	5.33 ± 0.33aB	5.39 ± 0.24aC	4.08 ± 0.43bC
70 °C	5.32 ± 0.32B	5.35 ± 0.23C	5.01 ± 0.33B
80 °C	8.36 ± 0.22aA	6.86 ± 0.24bB	5.57 ± 0.07cA
90 °C	8.23 ± 0.15aA	7.63 ± 0.13bA	6.08 ± 0.14cA

US, unsprouted sorghum flour; SSD50, flour from sprouted sorghum dried at 50 °C for 6 h; SSD40, flour from sprouted sorghum dried at 40 °C for 12 h; WHC, water-holding capacity; OHC, oil-holding capacity; Sp, swelling power; CU, ceralpha units; LOQ, limit of quantification Values are expressed as mean ± standard deviation (SD) (n ≥ 3; n = 2 for α-amylase activity). Different lowercase letters indicate significant differences (one-way analysis of variance (ANOVA) with Duncan’s post-hoc test, *p* ≤ 0.05) between different samples at the same temperature. For Sp, different capital letters indicate a significant difference (one-way ANOVA with Duncan’s post-hoc test, *p* ≤ 0.05) between temperatures for the same sample.

**Table 2:** Amino acid composition of sorghum flours expressed as g/100 g flour. In brackets, the % index of change compared to S value.

Amino Acid (g/100 g Flour)	US	SSD50	SSD40
Essential amino acids (EA)			
Histidine	0.193 ± 0.011	0.210 ± 0.005	0.204 ± 0.005
Isoleucine	0.372 ± 0.005 b	0.386 ± 0.007 ab (+3.76)	0.391 ± 0.010 a (+5.11)
Leucine	1.256 ± 0.021	1.232 ± 0.027	1.276 ± 0.029
Lysine	0.147 ± 0.002 c	0.153 ± 0 b (+4.08)	0.160 ± 0.001 a (+8.84)
Methionine	0.169 ± 0.011 b	0.174 ± 0.003 b (+2.96)	0.189 ± 0 a (+11.83)
Phenylalanine	0.550 ± 0.007	0.544 ± 0.005	0.561 ± 0.036
Threonine	0.315 ± 0.008	0.327 ± 0.002	0.328 ± 0.007
Valine	0.500 ± 0.011	0.476 ± 0.032	0.518 ± 0.010
Cysteine	0.188 ± 0.002 b	0.190 ± 0.005 b (+1.06)	0.198 ± 0.002 a (+5.32)
Tyrosine	0.243 ± 0.001	0.255 ± 0.002	0.260 ± 0.022
E/T (%)	43.5	43.2	42.7
Non-essential amino acids (NEA)			
Alanine	0.801 ± 0.020 ab	0.785 ± 0.010 b (− 2.00)	0.830 ± 0.018 a (+3.62)
Arginine	0.283 ± 0.014	0.289 ± 0.011	0.282 ± 0.007
Aspartic acid	0.586 ± 0.023 c	0.656 ± 0.008 b (+11.95)	0.707 ± 0.037 a (+20.65)
Glutamic acid	1.898 ± 0.103	1.861 ± 0.015	1.962 ± 0.066
Glycine	0.317 ± 0.004	0.303 ± 0.002	0.323 ± 0.014
Hydroxyproline	0.004 ± 0	0.005 ± 0.001	0.005 ± 0
Proline	0.782 ± 0 c	0.852 ± 0.023 b (+8.95)	0.908 ± 0.021 a (+16.11)
Serine	0.437 ± 0.002 b	0.447 ± 0.011 ab (+2.29)	0.464 ± 0.012 a (+6.18)
Total	9.040 ± 0.190 b	9.142 ± 0.142 b (+1.13)	9.564 ± 0.056 a (+5.80)

Values are expressed as mean ± SD (n = 3). Values followed by different letters in each column are significantly different (one-way ANOVA with Duncan’s post-hoc test, *p* ≤ 0.05). US, unsprouted sorghum flour; SSD50, flour from sprouted sorghum dried at 50 °C for 6 h; SSD40, flour from sprouted sorghum dried at 40 °C for 12 h.

## Data Availability

Data available on request.

## Supplementary Materials

The following are available online at https://www.mdpi.com/2304-8158/10/2/407/s1, Figure S1: Schematic representation of sample preparation. Figure S2: morphological observations of sorghum flours acquired with optical microscopy and number of cells per aggregate. Table S1: Thermal properties of unsprouted and sprouted sorghum flours.

## Abbreviations

US	Unsprouted sorghum flour
SSD50	Flour from sprouted sorghum dried at 50 °C for 6 h
SSD40	Flour from sprouted sorghum dried at 40 °C for 12 h
RH	Relative humidity
TDF	Total Dietary Fiber
IDF	Insoluble Dietary Fiber
SDF	Soluble Dietary Fiber
RS	Resistant starch
DS	Digestible Starch
TS	Total Starch
BSA	Bovine serum albumin
ΔH	Enthalpy
T_on_	Onset temperature
T_p_	Peak temperature
T_off_	Offset temperature
S_p_	Swelling power
R/T	Room temperature
SDS	Sodium dodecyl sulfate
β-ME	β-mercaptoethanol
DH	Degree of hydrolysis
UPLC	Ultra-performance liquid chromatography
ESI-MS	Electrospray ionization mass spectroscopy
SIR	Single Ion Recording
WHC	Water-holding capacity
OHC	Oil-holding capacity
BU	Brabender Units
LOQ	Limit of quantification
ESEM	Environmental scanning electron microscopy
DSC	Differential scanning calorimetry
SDS-PAGE	Sodium dodecyl sulphate-polyacrylamide gel electrophoresis
HMW	High molecular weight
M_r_	Molecular weight
ANOVA	Analysis of variance
AA	Amino acids
EA	Essential amino acids
NEA	Non-essential amino acids
TA	Total amino acids
