# Supplementary material for: Sprouting of Sorghum (Sorghum bicolor [L.] Moench): Effect of Drying Treatment on Protein and Starch Features
Source: Foods. 2021 Feb 12;10(2):407. doi: 10.3390/foods10020407 (PMC7917644; doi:10.3390/foods10020407)
Supplement: Supplementary file 1 [file foods-10-00407-s001.pdf]

Sprouting of Sorghum (*Sorghum bicolor* [L.] Moench): Effect of Drying Treatment on Protein and Starch Features

Mia Marchini [1], Alessandra Marti [2], Claudia Folli [1], Barbara Prandi [1], Tommaso Ganino [1,3], Paola Conte [4], Costantino Fadda [4], Monica Mattarozzi [5,6] and Eleonora Carini [1,6,*]

[1] Department of Food and Drug, University of Parma, Parco Area delle Scienze, 47/A, 43124 Parma, Italy; mia.marchini@unipr.it (M.M.); claudia.folli@unipr.it (C.F.); barbara.prandi@unipr.it (B.P.); tommaso.ganino@unipr.it (T.G.)
[2] Department of Food, Environmental, and Nutritional Sciences (DeFENS), University of Milan, Via G. Celoria 2, 20133 Milan, Italy; alessandra.marti@unimi.it
[3] Consiglio Nazionale delle Ricerche, Institute of BioEconomy (IBE), via Madonna del Piano 10, 50019 Sesto Fiorentino, Italy
[4] Dipartimento di Agraria, Sezione Scienze e Tecnologie Ambientali e Alimentari, Università degli Studi di Sassari, Viale Italia 39/A, 07100 Sassari, Italy; pconte@uniss.it (P.C.); cfadda@uniss.it (C.F.)
[5] Department of Chemistry, Life Sciences, and Environmental Sustainability, University of Parma, Parma, Italy, Parco Area delle Scienze 17/A, 43124 Parma, Italy; monica.mattarozzi@unipr.it
[6] Centro Interdipartimentale Sulla Sicurezza, Tecnologie e Innovazione Agroalimentare—SITEIA.PARMA, University of Parma, Via Università 12, 43121 Parma, Italy
* Correspondence: eleonora.carini@unipr.it; Tel.: +39-052-190-6520

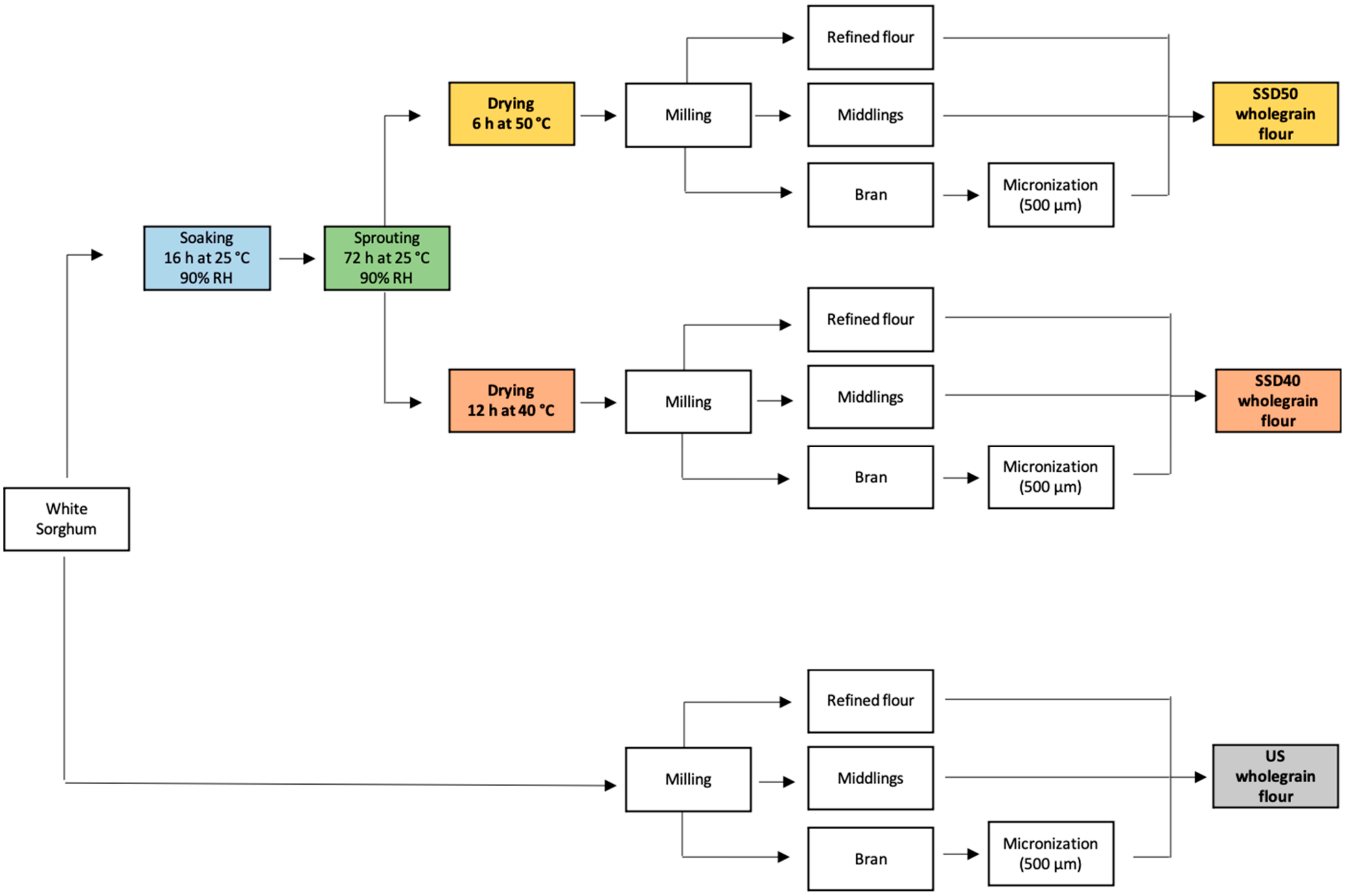

Figure S1. Schematic representation of sample preparation.

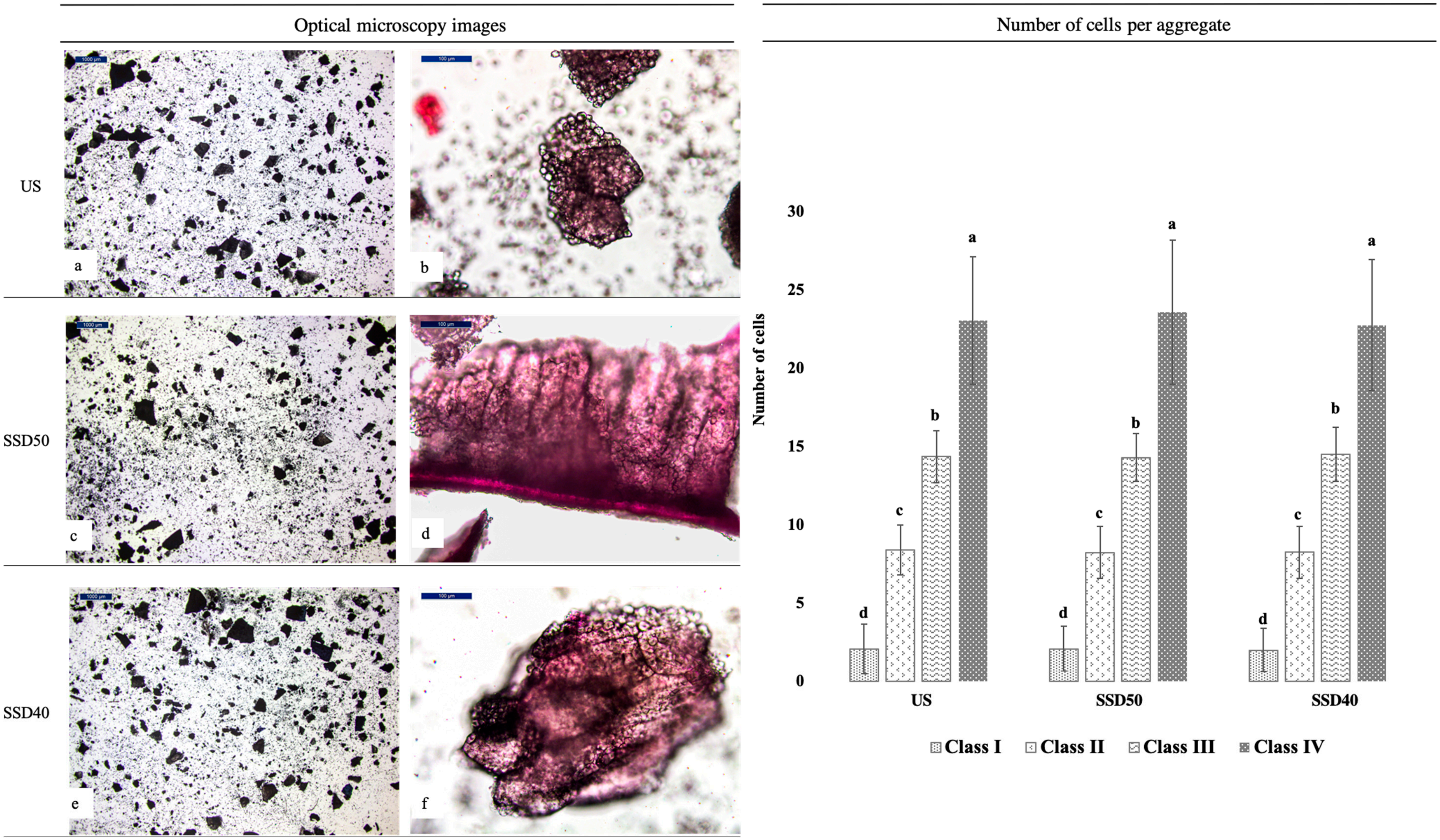

Figure S2. On the left, morphological observations of sorghum flours acquired with optical microscopy. Cell aggregates morphology (a, c, e; magnified 1.25x) and cells morphology (b, d, f; magnified 20x) in US, SSD50 and SSD40. On the right, number of cells per aggregate. For each sample, different letters mean statistically significant differences among the dimensional classes (one-way ANOVA and Duncan post-hoc) ($p < 0.05$). US, unsprouted sorghum flour; SSD50, flour from sprouted sorghum dried at 50 °C for 6 h; SSD40, flour from sprouted sorghum dried at 40 °C for 12 h.

Table S1. Thermal properties of unsprouted and sprouted sorghum flours.

	Starch gelatinization peak				Amylose – lipid complex peak			
	T_{on} (C°)	T_p (C°)	T_{off} (C°)	ΔH (J g^{-1})	T_{on} (C°)	T_p (C°)	T_{off} (C°)	ΔH (J g^{-1})
US	57.53 ± 0.72b	76.65 ± 0.1	91.3 ± 0.22a	2.3 ± 0.13a	94.92 ± 0.71	100.91 ± 0.02a	108 ± 0.28	0.23 ± 0.02b
SSD50	68.68 ± 0.55a	77.14 ± 0.29	85.97 ± 1.34b	1.64 ± 0.01b	95.95 ± 0.36	100.42 ± 0.78a	106.57 ± 0.48	0.22 ± 0.01b
SSD40	68.23 ± 0.18a	76.97 ± 0.16	86.12 ± 0.9b	1.63 ± 0.31b	93.7 ± 1.34	98.67 ± 4.65a	107.52 ± 0.93	0.33 ± 0.01a

Values are expressed as mean ± SD (n=3). US, unsprouted sorghum flour; SSD50, flour from sprouted sorghum dried at 50 °C for 6 h; SSD40, flour from sprouted sorghum dried at 40 °C for 12 h; To, onset temperature; Tp, peak temperature; Toff, offset temperature; ΔH, gelatinization enthalpy.
